# The Deese/Roediger–McDermott (DRM) illusion in short-term memory: Opposite effects of retention interval on true and false recognition

**DOI:** 10.3758/s13421-026-01881-7

**Published:** 2026-06-11

**Authors:** Guillermo Campoy, María D. Linde, Esteban A. Hidalgo, Miriam Tortajada, Lucía B. Palmero, Víctor Martínez-Pérez, Luis J. Fuentes

**Affiliations:** Faculty of Psychology and Speech Therapy, University of Murcia, Murcia, Spain

**Keywords:** False memory, DRM, Short-term memory, Working memory, Recognition, Retention interval, Fuzzy-trace theory, Explicit warnings, Bayesian multilevel modeling

## Abstract

A short-term memory (STM) version of the Deese/Roediger–McDermott (DRM) paradigm was employed to investigate how true and false recognition evolved as STM contents were lost over a short time window immediately after initial encoding. Presentations of five-word DRM lists were followed by list-specific recognition tests applied either immediately or after a distractor-filled retention interval of 3, 9, or 27 s. Results showed a decrease in the probability of true recognition and an increase in the probability of false recognition as the retention interval grew longer. Based on the fuzzy-trace theory, we suggest that this pattern emerged from the different durability of item-specific phonological representations, which would play a dual role of supporting true memory and preventing false recognition, and integrative semantic representations, whose overlap with the critical items would give rise to the DRM illusion. As a further contribution, our study helps to establish the DRM illusion in STM as a genuine and robust phenomenon by showing that it can be observed even with no delay and no intervening distraction between study and test, and that it occurs when participants are explicitly warned about the presence of semantically related distractors and instructed not to be misled by them.

## Introduction

Sometimes we have memories that do not correspond to actual past events. Besides practical implications, memory researchers are interested in this false memory phenomenon because it provides an important window into human memory processes. During the last decades, research in the field of false memory has been dominated by the Deese/Roediger–McDermott (DRM) paradigm (Deese, 1959; Roediger & McDermott, 1995), probably because of its simplicity, versatility, and robustness (Gallo, 2010). The typical procedure in DRM studies involves the presentation of word lists created based on free-association norms, so that each list consists of the strongest associates (e.g., *bed*, *rest*, *awake*, etc.) to a non-presented critical item (e.g., *sleep*). The basic finding is that, in subsequent memory testing, non-presented critical items are recalled or recognized at levels that approach or even exceed the recall or recognition of presented words (Gallo, 2006).

The two main accounts of the DRM illusion, not mutually exclusive, are the activation-monitoring theory (AMT; Roediger & McDermott, 2000; Roediger et al., 2001) and the fuzzy-trace theory (FTT; Brainerd & Reyna, 1998, 2002). According to the AMT, the key mechanism is the automatic spread of activation through associative-semantic networks. Because of this mechanism, the mental representation of the critical item becomes more and more activated as its associates are presented. Later, false recall or recognition occurs when monitoring processes (Johnson & Raye, 1981) fail to determine the real origin of that activation. In contrast, the FTT proposes that the DRM illusion results from the generation, during the encoding phase, of a gist representation that condenses items’ semantic content. Later, at the test phase, the consistency between critical items and gist traces leads to false recall or recognition unless a process of recollection rejection takes place. Recollection rejection involves the retrieval of the so-called verbatim traces, which are item-specific representations of the surface features of the list words that are generated at initial encoding in parallel with gist representations (Chang & Brainerd, 2021).

As noted by Roediger and Gallo (2022), although the AMT effectively accounts for numerous findings within the DRM paradigm, there is a specific phenomenon for which the explanation the FTT is better suited: the differential impact of the length of the retention interval on false and true memories. The reason for this is that, according to the AMT, once representations of critical items become activated, that activation is not qualitatively different from that of the presented items. If increasing the retention interval affects how the initial activation of an item is later translated into recall or recognition of this item, the length of the retention interval should similarly affect true and false memory. Thus the AMT does not predict differential effects of the length of the retention interval on true and false memory unless additional assumptions are made (Roediger & Gallo, 2022). The FTT, in contrast, easily accommodates the differential impact of retention intervals on true and false memory because performance hinges on two separate sets of representations, gist and verbatim traces, that exert opposite effects on the appearance of false memories and that may be differentially susceptible to the passage of time. If the availability and accessibility of gist and verbatim traces decline across retention intervals at different rates, then a differential effect of the length of the intervals on true and false memories naturally emerges. Given that our focus in the present study is on the impact of the retention-interval length on true and false memories, we adopt the FTT as the theoretical framework as its postulation of two separate sets of representations that may decline at different rates provides a direct mechanism to explain and predict differential effects of length.

Most previous DRM studies manipulating retention interval have employed DRM lists of about 12–15 items, a number well beyond the capacity of verbal short-term memory (STM; Miller, 1956). They also included, as their condition with the shortest interval, a memory test applied immediately after the presentation of all the experimental lists, habitually with the presentation of instruction or other minor arrangements between study and test phases (Seamon, et al., 2002). Because of these two features, it is reasonable to assume that performance in these studies relied exclusively on long-term memory (LTM). In the present study, however, we were interested in the alternative approach of employing shorter DRM lists and applying, for the condition with the shortest interval, a memory test immediately after the presentation of each list (Atkins & Reuter-Lorenz, 2008; Coane et al., 2007). This second approach could be especially fruitful for investigating the effect of verbatim traces across retention intervals because, unlike the more traditional approach, STM representations generated during the presentation of the items would still be available at the time of testing. STM representations possess two characteristics that may be key from the point of view of the FTT. First, according to current views, STM partly relies on the maintenance, via internal attention, of representations activated by perceptual inputs once these inputs have ceased (Ruchkin et al., 2003). A necessary consequence of this partial overlap between perceptual and STM representations is that STM representations should contain high levels of verbatim information. This would be the case of phonological representations, which are broadly assumed to play the main role in verbal STM tasks (Baddeley, 1966). Second, STM representations are in a state of high accessibility for the current mental task. This high-accessibility state is, in fact, the defining difference between STM and LTM representations, and is presumably the consequence of two underlying neural mechanisms, persistent neuronal firing and some form of short-term synaptic plasticity (Masse et al., 2020). Because of these two characteristics of STM representations, it seems reasonable to suggest that, in the context of a DRM task, the situation with the highest level of availability and accessibility of verbatim information would be that in which memory testing takes place when STM still maintains representations for all the just-presented items. The experiment described here investigated how true and false memory in this privileged situation of maximum availability and accessibility of verbatim information evolves as relevant STM contents are lost over time within a brief time window immediately following the list presentation. For that, we visually presented five-word DRM lists followed by a recognition test in which participants had to identify presented words from a set that included non-presented distractors and the critical item. The recognition test was applied either right after the presentation phase or after a retention interval of 3, 9, or 27 s. Retention intervals were filled with an attention-demanding distractor task that required participants to encode and temporally maintain numerical information, thereby competing for the same limited-capacity resources that would be needed to retain the list items in STM. Such a task thus served to prevent the use of maintenance mechanisms (i.e., articulatory rehearsal and attentional refreshing; Camos, 2015) and to promote the processing of interfering material. As a result, STM representations of the list items were expected to be progressively lost or displaced during the retention intervals due to decay (Barrouillet et al., 2004; Campoy, 2012) and/or interference (Campoy, 2011; Lewandowsky et al., 2009). Importantly, the characteristics of the recognition test and all other aspects of the procedure aside from the length of the retention intervals were kept equal across conditions to allow direct comparison.

To summarize, the present study examined how true and false recognition evolved from a condition in which STM representations for the presented items were still available thanks to the reduced number of items and the absence of a retention interval, to a condition in which the availability of these STM representations was largely diminished or even absent after 27 s of interfering activity. To the best of our knowledge, this is the first study to investigate the evolution of true and false memory during a brief time window immediately following the presentation of short DRM lists, which we believe contributes to characterizing and further understanding the phenomenon of the DRM illusion in STM and to reinforcing one of the core assumptions of FTT: that verbatim traces play a key role in protecting against false memories. Furthermore, our design allows for a direct comparison of the DRM effect across a temporal window that spans from STM to LTM. While some prior DRM studies have included both short- and long-term measures (Abadie & Camos, 2019; Flegal et al., 2010; Flegal & Reuter-Lorenz, 2014; Olszewska et al., 2015), a direct comparison in these precedents is challenging because the nature of the memory tests differed. The approach in these precedents was to contrast an (almost) immediate, list-specific recognition test with a delayed, global one pertaining to all the lists presented in the experiment. This important difference between measures makes a direct comparison questionable. The present study circumvents this methodological issue by employing an identical recognition test across all conditions, thus offering a more direct and comparable assessment of how the DRM effect evolves from STM to LTM. Additionally, this study may provide initial insights into the differential durability and/or susceptibility to interference of phonological traces compared to the integrative semantic representations that presumably underlie the DRM illusion in verbal STM. We will discuss the nature of these integrative semantic representations in the Discussion section. Finally, in the experiment reported here, participants were explicitly warned that the recognition test might include words that were semantically related to, but not actually presented among, the studied items, and that they should be careful not to be misled by them. To our knowledge, this is the first short-term DRM study to incorporate such a warning, which may help to assess the robustness of the illusion in the STM domain.

## Method

### Participants

A total of 190 undergraduate students from the University of Murcia (39 men, 151 women; *M*_*age*_ = 20.5 years, *SD* = 1.6) participated in the experiment, which was conducted online. All were native Spanish speakers and received course credit for their participation. Participants were recruited from a pool of students who had voluntarily enrolled in a research participation system administered by the Faculty of Psychology at the University of Murcia. Data from 27 additional participants were collected but excluded from the analyses for various reasons. Specifically, ten participants were excluded because they either did not complete all trials or completed the task more than once. In addition, data from seven participants were excluded for not being native Spanish speakers. Finally, data from ten participants were excluded due to anomalous performance patterns (e.g., extremely low accuracy in the distractor task; details regarding these performance-based exclusions are provided in the Results section).

The sample size was not determined via a priori power analysis, as we did not plan to adopt a frequentist approach for data analysis (we used Bayesian estimation). Conducting the experiment online allowed us to collect data from as many participants as possible. This larger sample helps offset the reduced experimental control typically associated with online settings and allows for more precise parameter estimates (i.e., narrower credible intervals). Note that, while in traditional frequentist statistics a large sample size can lead to statistically significant but practically trivial effects due to increased power, this is not a concern in Bayesian estimation. In this framework, conclusions are not based solely on whether there is a credible difference between conditions (i.e., the credible interval for the difference does not include zero), but also on the qualitative evaluation of the practical or theoretical meaningfulness of the values within the credible interval (Kruschke, 2018).

### Materials

We employed 24 newly constructed DRM lists. Each list consisted of a critical item and five to-be-presented words (list words), all associated with the critical item according to free-association norms. For example, the DRM list for the critical item *tree* (*árbol*) included the words *branch*, *pine*, *squirrel*, *forest*, *leaves* (*rama*, *pino*, *ardilla*, *bosque*, *hojas*). Additionally, each list included two less strongly associated words (*garden*, *park*; *jardín*, *parque*) to be used as related distractors and two unrelated words (*cover*, *wizard*; *portada*, *mago*) to be employed as unrelated distractors. These two types of distractors served as complementary baselines for estimating true and false recognition across intervals. As detailed below, related distractors were selected similarly to the list words (based on their associative strength with the critical item), whereas unrelated distractors were chosen using roughly the same selection criteria (i.e., familiarity, concreteness, imageability, frequency, number of syllables, grammatical category) as the critical items. Importantly, the inclusion of related distractors during the recognition test was also intended to discourage participants from adopting a simplistic, gist-based rejection strategy during the recognition test (McBride et al., 2019).

To construct the lists, we first selected a pool of 518 potential critical items from the EsPal database (Duchon et al., 2013). All selected words were nouns consisting of two to four syllables and met the following criteria: a log-transformed frequency (log_10_ of frequency per million +1) between 3.5 and 5.5; familiarity ratings between 5.5 and 7; imageability ratings between 4.5 and 7; and concreteness ratings also between 4.5 and 7 (the familiarity, imageability, and concreteness values were based on subjective ratings using 7-point Likert scales, where higher scores indicate greater levels). These selection criteria were established to reduce heterogeneity among critical items.

Subsequently, we used the University of Salamanca Spanish Free Association Norms Database (Díez et al., 2018) to identify associates for each potential critical item. Forward and backward association data were available for 298 of the initial 518 items, but only 101 had at least seven associates (note that we needed five associates as list words plus two associates as related distractors). From this reduced pool, we constructed as many lists as possible based on the following two criteria. First, no word could appear in more than one list. Second, the associates of a given critical item could not belong to the same lexical family or be phonologically similar to the critical item or to any other word in the list. Additionally, care was taken to ensure that critical items were unrelated to one another. Applying these constraints, we were able to construct an initial set of 30 lists. For each list, the five associates with the highest backward association values were selected as list words, and the following two associates were used as related distractors.^1^ The two unrelated distractors for each list were selected from the remaining items in the potential critical item pool, ensuring they were neither phonologically similar to nor semantically associated with any other word in the list.

The effectiveness of the 30 constructed lists was evaluated in a pilot study involving 48 participants. This study followed a simplified version of the procedure used in the main experiment described below. Based on the results, we selected 24 lists that reliably elicited false recognition of the corresponding critical items, 20 to be used as experimental lists and another four for the practice block. For the 20 experimental lists, the mean backward associative strength (BAS) from each list word to its corresponding critical item was .237 (range: .037–.486), and the mean forward associative strength (FAS) from the critical item to its list words was .048 (range: .007–.183). The related distractors had a mean BAS of .036 and a mean FAS of .029.

The experiment was implemented as a web application using custom-made code written in HTML, CSS, JavaScript, PHP, and SQL. We did not use any external libraries, frameworks, or toolkits to maintain full control over the program’s behavior and ensure maximum compatibility. Data were stored securely on servers maintained by the University of Murcia. Participants accessed the application using their institutional email addresses, which enabled us to verify participant identity, and completed the experiment on their own devices (e.g., computers, tablets, or smartphones). Special attention was paid to ensuring consistent visual appearance across different devices and browsers, achieved through extensive CSS styling resets (i.e., neutralizing default style variations), the use of locally hosted fonts, and a responsive design that dynamically adjusted element sizes to maintain visual proportionality.

### Procedure

Participants were instructed to complete the experiment in a quiet, distraction-free environment. They first completed a practice block of the distractor task to become familiar with it. In each trial of this task, a three-digit number was presented in written form, accompanied by four buttons, each displaying a different three-digit number in Arabic numerals. Participants were asked to click on the number that matched the written target as quickly as possible while minimizing errors. Target numbers were randomly selected without replacement from a pool of 588 three-digit numbers whose Spanish orthographic forms contained between 19 and 27 characters (including spaces). In each trial, the three distractors were generated by randomly modifying the target number by either increasing or decreasing one of its digits by one, or by permuting the order of its digits. These transformations produced distractors with high similarity to the target, thereby increasing the task's cognitive demands. Because participants had to hold a phonological representation of the written number in mind while selecting the correct option among similar alternatives, this task was expected to interfere with maintenance operations that might otherwise be used to retain the list items in STM.

After ten practice trials of the distractor task, participants proceeded to the main experiment. Each trial began when the participant pressed a start button. A 1,000-ms blank screen was followed by a fixation cross (800 ms on, 200 ms off). Then, the five list words were presented sequentially in uppercase letters at a rate of one word per second (800 ms on, 200 ms off). The order of the list words was randomized on each trial. In the condition with a 0-s retention interval, participants received the recognition test immediately after the final list item. In the other three conditions, participants completed the distractor task for 3, 9, or 27 s. These intervals were chosen to span a logarithmic progression, not based on a priori theoretical expectations, but only because many psychological phenomena tend to evolve logarithmically over time. When necessary, the final trial of the distractor task was truncated to match the duration of the retention interval.

The recognition test consisted of eight words displayed simultaneously as buttons arranged in two columns of four. These included the critical lure, two related distractors, two unrelated distractors, and three old items randomly selected from the five words presented in the study list. All words were randomly assigned to button positions. The number of old items presented in the recognition test was fixed at three based on a preliminary, unpublished study in which the number of old items varied between one and five (Campoy et al., 2025, Version 2). Results from that study showed that false recognition was higher when fewer old items were included in the test. However, this tendency disappeared when at least three list items were present, suggesting the possibility that limiting the number of old items to fewer than three may artificially inflate false recognition. During recognition, participants were allowed to select and deselect words freely until they clicked the *Finish* button to submit their final response. It is important to note that presenting all eight words simultaneously at recognition allowed participants to rapidly scan all alternatives and base their decisions on a global assessment of the test set. This contrasts with the alternative, slower procedure of presenting items sequentially, where recognition judgments can be influenced by the sequential order of stimuli. Moreover, a sequential procedure would severely distort the intended retention intervals by adding variable delays depending on each item’s random test position and the time taken to respond to preceding items.

Participants were explicitly informed that some of the distractors would be similar or related to the list words, in a way that could lead them to erroneously think these words had been presented. They were instructed not to be misled by that fact and to click only on the words they genuinely believed had appeared.

**Fig. 1:**
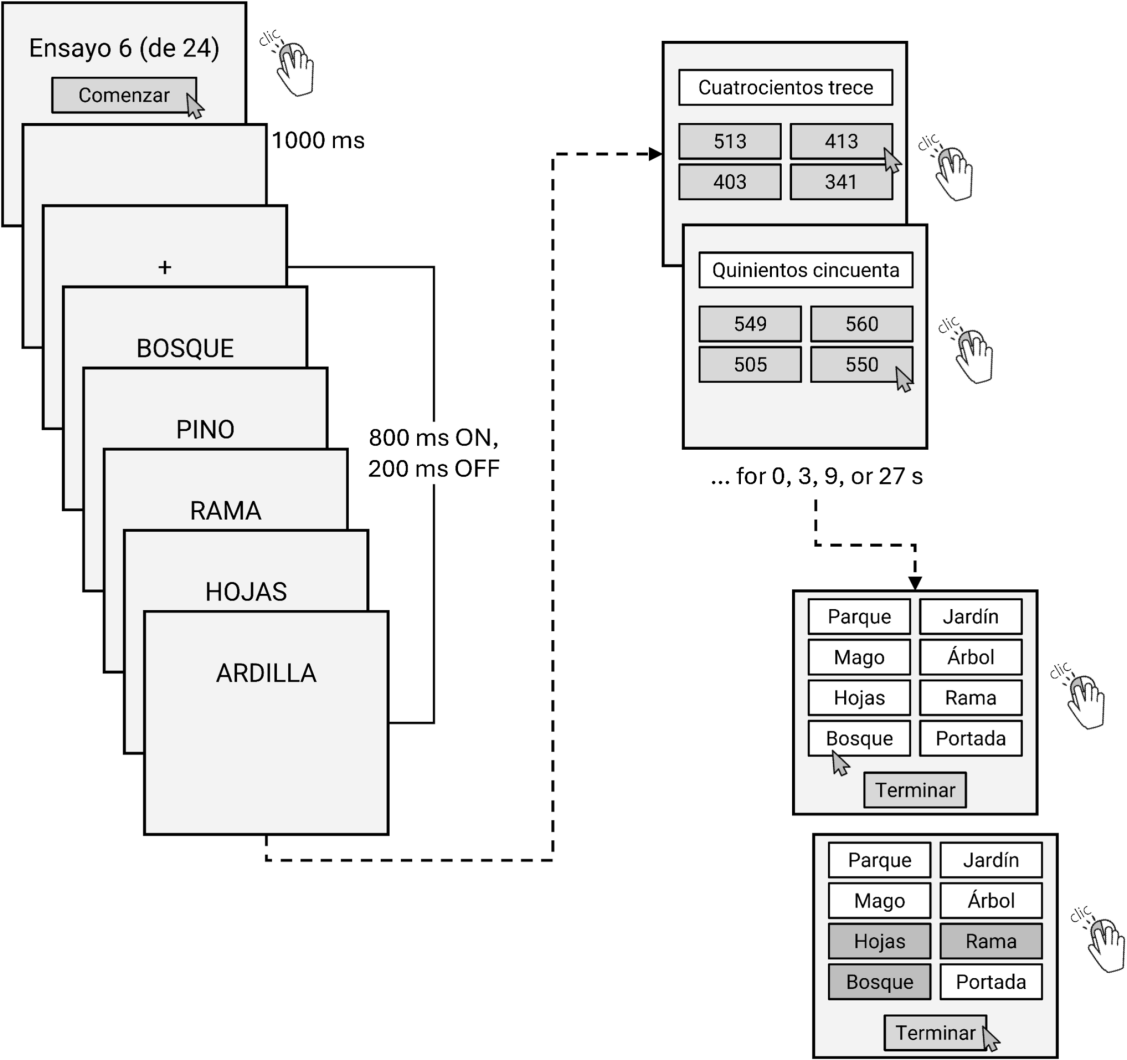
Schematic representation of the experimental procedure

Following four practice trials (one per retention interval), participants completed the experimental phase, which included 20 experimental trials, five per retention-interval condition. The 20 DRM lists were randomly assigned to the different retention intervals for each participant. The order of trials was randomized, with the constraint that there was one trial for each retention interval condition every four trials. A schematic overview of the procedure is provided in Fig. 1.

## Results

We first analyzed each participant’s individual performance to detect anomalous response patterns. This step is especially important in online experiments, where participants complete the task without supervision, potentially leading to less diligent engagement or greater susceptibility to external distractions compared to a laboratory setting. As a general criterion, we considered values exceeding three standard deviations from the group mean as outliers. We identified four participants with extremely low accuracy in the distractor task (z-scores = −5.95, −5.30, −3.79, and −3.10; the next lowest was −2.60). Moreover, three participants showed unusually high recognition rates for unrelated distractors (z-scores = 5.09 in all three cases; the next highest was 2.38). Another three participants exhibited exceptionally high recognition rates for related distractors (z-scores = 5.91, 5.24, and 4.58; the next highest was 2.59). Finally, one participant had an unusually low rate of true recognition (z = −3.38; the next lowest was −2.76). Since some met more than one exclusion criterion, the process resulted in the identification of only ten participants with anomalous performance; their data were excluded from the analyses.

As anticipated earlier, we adopted a Bayesian estimation approach (McElreath, 2020) for data analysis. The software used was R 4.3.2 (R Core Team, 2023), the brms 2.23.0 package (Bürkner, 2017), and Stan 2.33.1 (Stan Development Team, 2019).

We began by fitting a multilevel binomial regression model that predicted the probability of recognition of each test item as a function of type of item (list word, critical item, related distractor, or unrelated distractor) and retention interval (0, 3, 9, or 27 s). All these predictor variables were treated as categorical. The model included varying intercepts for both participants and stimuli, with stimuli nested within item type. This allowed us to account for individual differences between participants in their overall tendency to provide recognition responses, as well as for differences between stimuli in their propensity to elicit such responses. Weak informative priors were adopted for all parameters. Such priors facilitate model convergence and improve computational efficiency by guiding the sampling algorithm toward a broad range of plausible parameter values, while ensuring that the final estimates are driven by the observed data (Kruschke, 2018).

Posterior distributions were estimated using Markov Chain Monte Carlo (MCMC) sampling. Estimates were derived from 12,000 samples, obtained after 12,000 warm-up interactions. Model convergence was assessed by inspection of the trace plots, the R-hat values, and the number of effective samples. No convergence problems were detected, with all R-hats = 1.00. Bayesian estimates are probability distributions, denominated posterior distributions, that represent the probability or credibility of each potential value given the model and the data. Table 1 summarizes these posterior distributions with the mean and the 97% highest posterior density interval (HPDI), the narrowest interval containing 97% of the posterior probability and including the most probable (credible) values (all HPDIs reported below are also 97% intervals, even when the percentage is not explicitly stated). Table 1 also includes the empirical means (i.e., the proportions of recognition observed in the raw data) to facilitate comparison between observed and model-estimated values.^2^

**Table 1:** Bayesian estimates of the probability of recognition and empirical means

Type of item	Retention interval	Posterior *M*	97% HPDI	Empirical *M*
Critical item	0 s	.117	[.084, .154]	.121
	3 s	.179	[.134, .229]	.200
	9 s	.218	[.166, .275]	.237
	27 s	.262	[.200, .323]	.281
List word	0 s	.939	[.926, .950]	.934
	3 s	.886	[.867, .905]	.871
	9 s	.836	[.812, .861]	.817
	27 s	.814	[.788, .840]	.796
Unrelated distractor	0 s	.002	[.001, .004]	.002
	3 s	.001	[.000, .002]	.001
	9 s	.003	[.001, .005]	.003
	27 s	.003	[.001, .006]	.004
Related distractor	0 s	.010	[.006, .015]	.012
	3 s	.017	[.011, .024]	.021
	9 s	.024	[.016, .033]	.031
	27 s	.026	[.017, .035]	.032

*Note:* Posterior *M* = mean of the posterior distribution; HPDI = highest posterior density interval; Empirical *M* = mean of the observed data (i.e., proportion of recognition responses)

After fitting the model, we calculated bias-corrected probabilities of true and false recognition using both related and unrelated distractors as baselines (Chang & Brainerd, 2021). The bias-corrected false recognition score was defined as the difference between the probability of recognizing critical items and that of recognizing distractors. Similarly, bias-corrected true recognition was calculated as the difference between recognition of list words and distractors. Although the probability of recognizing related distractors was higher than that of unrelated distractors (difference collapsing across intervals: *M* =.017, 97% HPDI [.012, .022]; note also the non-overlapping credibility intervals at each retention interval in Table 1), the pattern of results was equivalent regardless of which distractor type was used as a baseline.

For the 0-s condition, which serves as the reference point for analyzing how recognition changes over time, the bias-corrected probability of false recognition was *M* =.107, HPDI [.074, .143] when using related distractors, and *M* =.115, HPDI [.082, .151] when using unrelated distractors. An estimated bias-corrected probability of at least.07 in the condition with no retention interval suggests a reliable DRM effect in the STM domain. For true recognition, the corresponding values were *M* =.929, HPDI [.916, .941] with related distractors and *M* =.937, HPDI [.925, .949] with unrelated distractors.

**Fig. 2:**
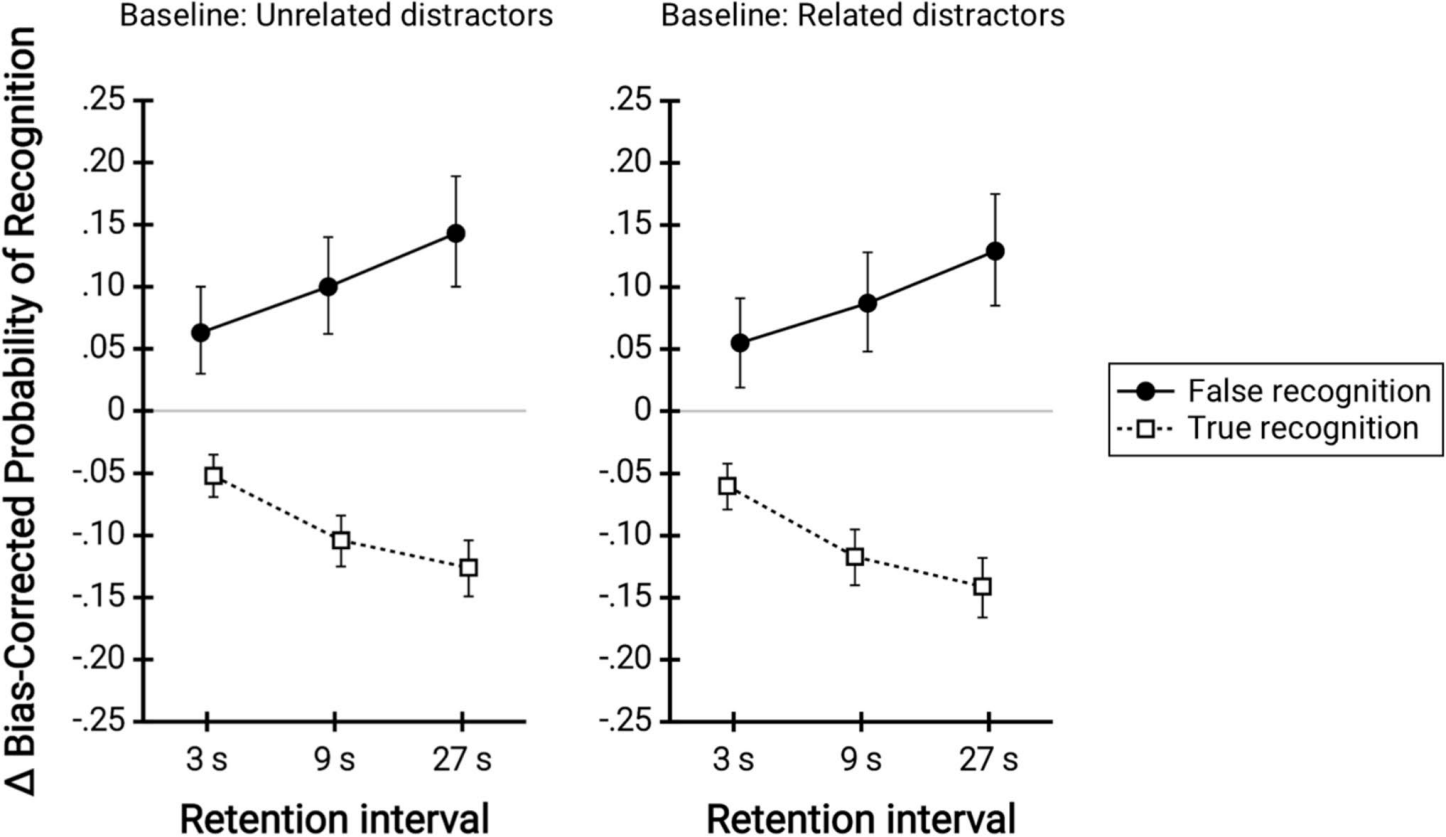
Estimates (posterior means and 97% HPDIs) of the change (Δ) in bias-corrected probability of true and false recognition relative to the 0-s condition. The x-axis is on a logarithmic scale

Figure 2 shows how bias-corrected scores evolved across retention intervals, expressed as differences relative to the 0-s condition (see also Table 2 for a comprehensive assessment of the differences between all conditions). Inspection of these estimates reveals a clear pattern. On the one hand, the probability of false recognition increased progressively with longer retention intervals. Notably, this increase was already evident at the 3-s interval, in which the lower bound of the HPDI for the change in bias-corrected false recognition relative to the 0-s condition was about .02, representing a relative increase of at least 13%. Interestingly, once a retention interval has been introduced, the growth in false recognition as this interval increases appears to follow a roughly linear trend when plotted on a logarithmic scale, suggesting a logarithmic relationship between interval duration and DRM illusion. As we only tested three non-zero intervals (3, 9, and 27 s), however, any conclusion about the precise form of this relationship should remain tentative. On the other hand, true recognition showed a decreasing trend across retention intervals, roughly mirroring the increase in false recognition. To assess whether the rates of change differed meaningfully between the two types of recognition, we directly compared the magnitude of the decrease in bias-corrected true recognition to the magnitude of the increase in bias-corrected false recognition at each interval. The results showed that the mean differences were small and the HPDIs clearly included zero in all three comparisons, indicating no credible difference in the overall rate of change between true and false recognition. Specifically, for retention intervals of 3, 9, and 27 s, respectively, posterior means for the difference and 97% HPDIs were −.005 [−.046, .038], −.030 [−.076, .020], and −.012 [−.062, .043] when related distractors were used as the baseline; and .011 [−.028, .051], −.004 [−.047, .044], and .017 [−.035, .067] with unrelated distractors as the baseline.

**Table 2:** Bayesian estimates of pairwise differences in bias-corrected false and true recognition probabilities across retention intervals using unrelated and related distractors as baselines

Recognition	Baseline	Comparison	Posterior *M*	97% HPDI
False	Unrelated	0 vs. 3 s	.063	[.030, .100]
		0 vs. 9 s	.100	[.062, .140]
		0 vs. 27 s	.143	[.100, .189]
		3 vs. 9 s	.037	[-.002, .078]
		3 vs. 27 s	.080	[.036, .124]
		9 vs. 27 s	.043	[.002, .089]
	Related	0 vs. 3 s	.055	[.019, .091]
		0 vs. 9 s	.087	[.048, .128]
		0 vs. 27 s	.129	[.085, .175]
		3 vs. 9 s	.032	[-.009, .073]
		3 vs. 27 s	.074	[.031, .120]
		9 vs. 27 s	.042	[-.000, .089]
True	Unrelated	0 vs. 3 s	-.052	[-.069, -.035]
		0 vs. 9 s	-.104	[-.125, -.084]
		0 vs. 27 s	-.126	[-.149, -.104]
		3 vs. 9 s	-.051	[-.072, -.031]
		3 vs. 27 s	-.074	[-.096, -.053]
		9 vs. 27 s	-.023	[-.045, -.000]
	Related	0 vs. 3 s	-.060	[-.079, -.042]
		0 vs. 9 s	-.117	[-.140, -.095]
		0 vs. 27 s	-.141	[-.166, -.118]
		3 vs. 9 s	-.057	[-.079, -.035]
		3 vs. 27 s	-.080	[-.103, -.058]
		9 vs. 27 s	-.024	[-.048, .000]

*Note:* Posterior *M* = mean of the posterior distribution; HPDI = highest posterior density interval

## Discussion

According to traditional views, verbal STM relies on the encoding and maintenance of phonological representations (Baddeley & Hitch, 1974). This view emerged from the observation that some phonological characteristics of the presented memoranda have conspicuous effects on STM performance, as occurs in the phonological similarity effect (Conrad & Hull, 1964) and the word-length effect (Baddeley et al., 1975). Moreover, when participants produce an extra-experimental word during immediate serial recall of lists of unrelated words, this word is usually a phonological neighbor of one presented item (Hulme et al., 1997), further supporting the reliance on phonological representations. In the last decades, however, there has been a growing interest in the effects of semantic factors such as word concreteness/imageability (Walker & Hulme, 1999) and semantic relatedness (Poirier & Saint-Aubin, 1995) on STM, leading to the current dominant notion that verbal STM relies on both phonological and semantic representations, with semantic effects being explained by interactive processes between and within these different representation levels (Castellà & Campoy, 2018; Kowialiewski & Majerus, 2020; Savill et al., 2017; Schwering & MacDonald, 2020). The fact that STM is susceptible to the DRM illusion (Atkins & Reuter-Lorenz, 2008; Coane et al., 2007) may represent additional evidence for the participation of semantic representations in verbal STM.

To further characterize and understand the DRM illusion in STM, the present experiment evaluated how true and false recognition evolved within a short time window immediately following item presentation. We employed DRM lists shorter than in standard DRM procedures (five items instead of about 12 or 15) and included a condition without a retention interval to estimate the probability of true and false recognition when presumably STM still holds representations for all the presented words at the time of testing. In other conditions, there were retention intervals (filled with distractor activity) of 3, 9, or 27 s while all other experimental parameters were kept equal. Consequently, we could estimate how the probability of true and false recognition changed as STM contents were progressively lost. The results were clear-cut: the probability of true recognition decreased, and the probability of false recognition increased as the retention interval grew longer.

The FTT provides a suitable framework to articulate an interpretation of the present results grounded on our knowledge of verbal STM. In an STM adaptation of the FTT, phonological traces are the obvious candidates to act as verbatim traces, playing the dual role of promoting true memory and preventing the DRM illusion. In our study, phonological representations of the presented items had the highest level of availability and accessibility just after the presentation phase, which explains why the highest probability of true recognition and the lowest probability of false recognition appeared in the condition without a retention interval. As the recognition test was delayed and participants performed the distractor task for longer, phonological representations progressively degraded through decay, retroactive interference, or both, having opposite effects on the probability of correct and false recognition. Note that the use of phonological representations to reject a critical item can be seriously hampered if one or more list items are phonologically similar to this critical item (McBride et al., 2019). That is why we explicitly avoided this kind of similarity (see *Materials*).

Turning to the role of gist traces, this may be played by the kind of representations that result from the operation of the conceptual STM system proposed by Potter (2012, 2018). According to Potter, when we perceive a meaningful stimulus such as a word, conceptual information and other associated contents are quickly activated from LTM. When a meaningful pattern or structure is identified among all concurrent active information, new links are rapidly formed in a process that ends with the generation of a conscious representation of the general meaning of the information. Conceptual STM is for example responsible for the fact that we are effortlessly aware of the meaning of a sentence immediately after its presentation (Potter & Lombardi, 1990). With the presentation of lists of unrelated words, however, there is no meaningful structure to build, so immediate memory depends exclusively on standard STM representations. The fact that an integrative conceptual representation is available after the presentation of a sentence but not after a list of unrelated words could partly explain why participants in STM tasks recall many more words when they are asked to recall sentences than when they must recall unrelated words (Allen et al., 2018). It would also explain why, in sentence recall, semantic errors (e.g., misrecalling *carpet* instead of *rug*) were more frequent than phonological errors, contrary to what occurs with lists of unrelated words (Jefferies et al., 2004). Based on these notions, a first tentative suggestion is that false recognition in our study resulted from the correspondence between critical items and gist representations generated by conceptual STM after structuring the semantic and conceptual information activated by DRM lists.

A more recent proposal within the STM literature is that, when lists of semantically related words are presented for immediate recall, participants can group information into supra-item semantic representations to reduce STM load during encoding and maintenance (Kowialiewski & Majerus, 2020). This mechanism of information compression in STM has been suggested to explain why immediate item memory is better for lists of semantically related words than for lists of unrelated words (Kowialiewski & Majerus, 2020; Kowialiewski et al., 2022b), although other explanations have been put forward (Kowialiewski et al., 2022a). Based on that, a second proposal regarding gist traces in verbal STM is that such a compression mechanism also operates on DRM lists, with critical items, on which all list words converge, emerging as suitable supra-item representations for their corresponding lists. In either case, our results support the idea that the availability and accessibility of this kind of integrative semantic representation decreased less over time with distraction than the phonological traces.

Regarding precedents of our research, this study represents, to the best of our knowledge, the first attempt to evaluate the evolution of true and false recognition during the first seconds immediately after the presentation of short DRM lists. A particularly relevant precedent is the early study by McDermott (1996), in which participants were asked to freely recall DRM lists either immediately after their presentation or after a 30-s retention interval filled with math problems. Results showed poorer veridical recall on the delayed measure, but no difference between interval conditions on false recall. That study, however, differs importantly from the present one in that McDermott’s lists consisted of 15 items, a number far beyond the capacity of verbal STM, so that performance in her immediate condition greatly depended on LTM (Glanzer & Cunitz, 1966). Most recently, and focused on a wider temporal window than our study, several DRM studies with short lists have evaluated false recognition by both an almost immediate test alleged to tap STM (a list-specific recognition test applied after an interfering activity of about 3 s) and a global LTM recognition test applied a few minutes after the presentation of all the lists (Abadie & Camos, 2019; Flegal et al., 2010; Flegal & Reuter-Lorenz, 2014; Olszewska et al., 2015). These studies consistently reported better true recognition in the immediate test but failed to find consistent evidence regarding false recognition in the delayed test. It must be noted, however, that immediate and delayed measures in these studies, besides the length of the retention interval, also differed in the fact that the search set in the immediate test only comprised the items just presented whereas the search set in the delayed measure consisted of all the words from all the lists presented. As anticipated in the Introduction section, this important difference between measures makes direct comparison questionable (for an equivalent argument, see McDermott, 1996). Here, we circumvented this issue by keeping all the aspects of the procedure equal, except for the length of retention intervals.

Moving to the LTM domain, some DRM studies have compared performance immediately after the presentation of all the DRM lists with measures obtained after longer intervals, typically 2 or 7 days. These LTM studies consistently reported a decline in true memory with longer intervals, but results regarding false recall or recognition of critical items exhibited much greater variability. The most common finding in recognition studies was that false recognition decreased over retention intervals to a similar extent to true recognition (Brainerd et al., 2001; Lampinen & Schwartz, 2000; Neuschatz et al., 2001; Seamon et al., 2002, Experiment 2; Sherman, 2013, Experiments 1B and 2B), suggesting that the length of the retention interval did not differentially affect true and false recognition. However, other similar studies observed that false recognition decreased over time to a lesser extent (Payne et al., 1996; Thapar & McDermott, 2001, Experiment 2), whereas the opposite pattern (greater decline of false recognition) has also been described (Colbert & McBride, 2007). To our knowledge, no recognition studies employing the necessary bias-corrected measures have found an increase of false recognition over time. On the other hand, recall studies found that either false recall decreased to a lesser extent than true memory (Thapar & McDermott, 2001, Experiment 1), did not decrease over time (Seamon et al., 2002, Experiment 1; Toglia et al., 1999), or even increased (Sherman, 2013, Experiments 1 A and 2 A), although this latter study employed non-standard DRM lists (brand names). It seems, therefore, that the increase in false memory over time that we observed in the present study is far from being a common finding in the long-term memory domain.

Besides the effect of the retention interval discussed so far, it is worth highlighting that our results show that false recognition of critical items can occur even when the memory test is administered immediately after list presentation, without any intervening distractor task. This finding is consistent with previous studies reporting false recognition under similar immediate testing conditions (Atkins & Reuter-Lorenz, 2008; Coane et al., 2007; Coane et al., 2024). It contrasts, however, with the suggestion by Abadie and Camos (2019) that false recognition in STM depends on phonological/verbatim traces being degraded by a brief distractor task that interferes with articulatory rehearsal during the first few seconds after list presentation. While our results do not support the idea that such distraction is necessary for the DRM illusion to emerge, they do align with the notion that even a short delay with interference can amplify the effect. Specifically, our study shows that just 3 s of distracting activity are enough to produce a clear increase in false recognition. This may have important implications for interpreting results from short-term false-memory studies that include a brief distractor task before testing (e.g., Abadie & Camos, 2019; Flegal et al., 2010; Flegal & Reuter-Lorenz, 2014; Olszewska et al., 2015), especially considering that findings from studies using both the retrocue paradigm (Oberauer, 2002, 2018; Tortajada et al., 2025) and the recent-probes tasks (Campoy, 2012) suggest that STM representations lose their highest level of accessibility (i.e., abandon the focus of attention) very rapidly (in less than 3 s) once attention shifts away from them to other information.

Finally, it is noteworthy that false recognition in the present study was found despite explicit warnings about the presence of critical items at recognition and instructions against being misled by them. LTM studies have shown that such warnings do not eliminate the DRM illusion (Neuschatz et al., 2001). The current findings indicate that this resistance to explicit warnings also applies to the STM domain, further underscoring the robustness of the DRM effect.

## Data Availability

The collected data and the stimuli used are available at Campoy et al. (2025): 10.5281/zenodo.16786375.

## Footnotes

1 An alternative procedure would have been to randomly select which associates served as list items and which as related distractors (e.g., Coane et al., 2024). We opted to use the strongest associates as list items to maximize the sensitivity of our task to detect changes in the DRM effect across retention intervals. As is well established, the magnitude of the DRM illusion depends on the number of items presented in the DRM lists (Robinson & Roediger, 1997). Given that the limited capacity of STM necessitated the use of short lists (five items in our case), we prioritized using the words presumably capable of generating the highest levels of false recognition. Another reason for departing from the procedure employed by Coane et al. (2024) is that they randomized the selection of associates for each participant, resulting in non-identical DRM lists across subjects. This approach was unsuitable for our study because we intended to leverage the flexibility of Bayesian multilevel models to include random intercepts not only for participants but also for items. This allows for statistical control over the varying potential of each list to generate the DRM illusion, representing a particularly relevant virtue of our data analysis approach. Consequently, we required the DRM lists to be identical for all participants, rendering randomization by participant inappropriate. It is important to note that, despite reserving the five primary associates as list items, the words used as related distractors were the next strongest in associative strength, remaining useful for discouraging a simplistic, gist-based recognition criterion. Examples illustrating that these distractors remain clearly related include *wings* and *cloud* for *airplane* (*alas* and *nube*, for *avión*), *actor* and *theater seat* for *theater* (*actor* and *butaca* for *teatro*), and *drink* and *thirst* for *drinking-glass* (*bebida* and *sed* for *vaso*).

2 Comparison of the values in Table 1 shows that the model estimates are slightly more conservative than the empirical means. This difference arises because the arithmetic mean is highly sensitive to the shape of the distribution. As a natural consequence of the fact that values cannot fall below 0 for false recognition or exceed 1 for true recognition, our data exhibited asymmetric distributions: the distribution for false recognition showed a longer tail towards higher values, while the distribution for true recognition showed a longer tail towards lower values. These extremes pull the arithmetic means away from the central tendency. In contrast, the Bayesian model is less influenced by such values. This reduced sensitivity to extreme values is, in fact, one of the main advantages of Bayesian multilevel modeling over the arithmetic mean when the goal is to accurately estimate the underlying central tendency.

## Code Availability

The analysis code is available at Campoy et al. (2025): 10.5281/zenodo.16786375.

## Declarations

### Preregistration

This study was not preregistered.
